# Hydroxyurea and Caffeine Impact pRb-like Protein-Dependent Chromatin Architecture Profiles in Interphase Cells of *Vicia faba*

**DOI:** 10.3390/ijms22094572

**Published:** 2021-04-27

**Authors:** Marcelina W. Musiałek, Joanna Deckert, Dorota Rybaczek

**Affiliations:** 1 Department of Cytophysiology, Institute of Experimental Biology, Faculty of Biology and Environmental Protection, University of Lodz, Pomorska 141/143, 90-236 Lodz, Poland; marcelina.musialek@edu.uni.lodz.pl; 2 Department of Plant Ecophysiology, Institute of Experimental Biology, Faculty of Biology, Adam Mickiewicz University Poznań, Uniwersytetu Poznańskiego 6, 61-614 Poznań, Poland; joanna.deckert@amu.edu.pl

**Keywords:** caffeine, cell cycle, chromatin architecture, 3D modeling, heterochromatin, hydroxyurea, plant, premature chromosome condensation, replication stress, retinoblastoma protein

## Abstract

The survival of cells depends on their ability to replicate correctly genetic material. Cells exposed to replication stress can experience a number of problems that may lead to deregulated proliferation, the development of cancer, and/or programmed cell death. In this article, we have induced prolonged replication arrest via hydroxyurea (HU) treatment and also premature chromosome condensation (PCC) by co-treatment with HU and caffeine (CF) in the root meristem cells of *Vicia faba*. We have analyzed the changes in the activities of retinoblastoma-like protein (RbS807/811ph). Results obtained from the immunocytochemical detection of RbS807/811ph allowed us to distinguish five unique activity profiles of pRb. We have also performed detailed 3D modeling using Blender 2.9.1., based on the original data and some final conclusions. 3D models helped us to visualize better the events occurring within the nuclei and acted as a high-resolution aid for presenting the results. We have found that, despite the decrease in pRb activity, its activity profiles were mostly intact and clearly recognizable, with some local alterations that may correspond to the increased demand in transcriptional activity. Our findings suggest that *Vicia faba’s* ability to withstand harsh environments may come from its well-developed and highly effective response to replication stress.

## 1. Introduction

*Vicia faba* is a crop plant widely cultivated all over the world. It is used as: (i) cover crop (for managing soil quality, fertility, erosion, water levels, weeds, pests, and overall biodiversity in an agroecosystem), (ii) a culinary ingredient, (iii) animal fodder, and (iv) a natural source of L-DOPA (a precursor of dopamine) used for curing patients with Parkinson’s disease [1]. Interestingly, it is also one of the easiest plants to grow. *Vicia faba* can withstand cold and harsh climates as well as grow in very dry or highly salinized soils. The ability to survive in demanding conditions and still provide value to humans (either culinary, agricultural, or medical) may soon become very valuable amid climate change and the increasing pollution of the planet.

Former studies performed by Rybaczek et al. [2,3,4] have shown that even under extremely stressful conditions, *V. faba* can manage stress well considering the induction of premature chromosome condensation (PCC)—an aberrant event well-known for its frequent initiation of genome chaos [5,6]. Under the conditions of replication stress caused by hydroxyurea (HU), which arrests replication by inhibiting the enzyme ribonucleotide reductase [7], the root meristem cells of the plant have shown incredible resistance to prolonged stressful conditions [4] and were able to recover fairly quickly once released into water [8]. Although PCC induction causes some chaotic aberrations in a fraction of cells, *V. faba* is mostly capable of directing the fraction of highly damaged cells to the apoptotic-like programmed cell death pathway (AL-PCD). The plant cells also recovered during the next cell cycle following PCC induction [4,8]. We believe that the high survivability rates of the *V. faba* family stem from the highly effective stress response mechanisms that are activated during replication and/or mitotic division. The identity of the cell must be maintained throughout the cell cycle in order to ensure the survival and growth of the organism. For this to occur, the nucleosomal organization and genetic information must be replicated into the daughter cells. With this in consideration, we suppose that the replication process and the mechanisms guarding its integrity and progress play a pivotal role in *V. faba’s* environmental resilience.

The most essential process for the reproduction and growth of eukaryotic organisms is the cell cycle [9], and many of the pathways that regulate the transitions between the consecutive subphases are conserved between eukaryotic species [10,11]. The transition from the G1 to the S phase is one of the most crucial steps during cell life as it determines the cell’s readiness for division [12]. Retinoblastoma protein (pRb) acts as the main regulator of the progression from the G1 to the S phase [13,14] in both animals and plants [15,16]. Hypophosphorylated pRb, through its binding with the E2F protein family, acts as a negative regulator of G1/S progression [17]. It retains the closed conformation structure of the chromatin in the regions where E2F-regulated genes are located [16,17]. The E2F family consists of transcriptional factors that either promote transcription (i.e., E2F1, E2F2, or E2F3a) or repress it (like E2F3b for instance). Some of them can act as both repressors and activators [18]. Retinoblastoma protein is phosphorylated by the complexes of cyclin and cyclin-dependent kinases (for instance, CYCD/E with CDK4/2 in humans), which changes its structure and results in the release of E2F [19,20,21,22,23]. In general, pRb phosphorylation leads to the expression of E2F-related genes as well as alterations in the recruitment of chromatin remodeling factors [13,24], allowing for the start of the S-phase and replication.

Studies show that RB-related regulatory pathways are highly conserved among eukaryotic organisms [13,14,15,16,25,26], and the retinoblastoma-related (RBR) plant ortholog follows the same regulatory pathway as pRb in humans [20,27,28]. The deregulation of the pRb-related pathway leads to the alteration of the cell cycle [29] and may result in many disturbances such as DNA damage, mitotic errors, or alterations in chromatin condensation [30,31,32,33]. The deregulation of pRb phosphorylation (especially its hyperphosphorylation) usually leads to excessive proliferation and is a common trait in many types of cancer [13,17,34,35]. For this reason, many studies suggest the pRb pathway as a target in alternative cancer treatments [35].

Retinoblastoma protein generally regulates the cell cycle by modifying chromatin structure [31,36,37]. Chromatin constitution controls its accessibility, and various remodeling factors have been demonstrated to interact with pRb [31]. For instance, the SWI/SNF complex remodels chromatin in an ATP-dependent manner by exchanging or removing histones from DNA (this pathway controls the general chromatin constitution). ATP-dependent histone exchange/removal complex (SWI/SNF) has also been reported to interact with pRb [38]. Retinoblastoma protein may also use the ATPase activity of Brm or BRG1 [39]. It frequently regulates local chromatin structures by modulating the balance of histone acetylation levels via recruitment of HDAC1 [13,40]. Histone acetylation opens the structure of the chromatin and facilitates transcription. Apart from local nucleosome structure regulation, pRb also plays a vital role in heterochromatin formation and segregation of mitotic chromosomes [31]. It binds to Suv39h and some members of the HP1 protein family as well as to methyltransferases that trimethylate histone H4k20 [41]. Retinoblastoma protein has been reported to regulate the pericentromeric heterochromatin [42] and telomeres [43] via histone methylation. Its inactivation thus leads to an aberrant chromatin structure [30,32,44,45] and is associated with chromosomal instability [46].

Changes in chromatin structure, both local and global, generally accumulate in cancer cells. As mentioned previously, deregulation of pRb leads to the overexpression of S-phase genes and significant changes in the heterochromatin constitution. Additionally, replication stress may challenge histone recycling and has been reported to accumulate ssDNA at replication sites, disrupting the nucleosomes in the process [47,48,49]. Hydroxyurea- (HU-) induced replication arrest causes severe under-replication in heterochromatin areas. This event is especially dangerous due to the fact that stalled replication forks within heterochromatin require ATRX factor for additional protection [50]. As well as this, the degree of chromatin condensation has a significant impact on the DNA damage response in the nucleus [51], but the mechanisms responsible for the reorganization and their implications are yet to be studied.

In this research, we focused on the changes in the activity of RbS807/811ph (diphosphorylated form of Rb-like protein present in plants) in *Vicia faba* root meristem cells subjected to prolonged replication stress (caused by hydroxyurea-induced replication arrest) as well as with the induction of premature chromosome condensation (PCC)—an event usually leading to genome chaos. We have performed an extended quantitative image analysis together with 3D modeling in order to analyze the impact of HU and PCC on the activity and behavior of pRb. We were able to distinguish five different activity patterns in interphase cells related to different transcriptional activity demands. We have shown an active response of cells subjected to replication stress and, particularly, PCC occurring in the perinucleolar area of chromatin, which most probably impacts the future well-being of the cells. We have also found that, despite the loss in the overall activity of pRb, its activity profiles are mostly undisturbed and still clearly recognizable after HU and PCC treatment, which shows that the response to the stressors is still properly ordered in analyzed cells. In our opinion, the mechanisms that respond to replication stress and, among other things, reorganize heterochromatin, play a pivotal role in cell survival and maintaining its integrity.

## 2. Results

### 2.1. The Effects of Prolonged HU and HU/CF Incubation on Heterochromatin Areas, Replication, and the Activity of CycD1 and RbS807/811

**Figure 1:**
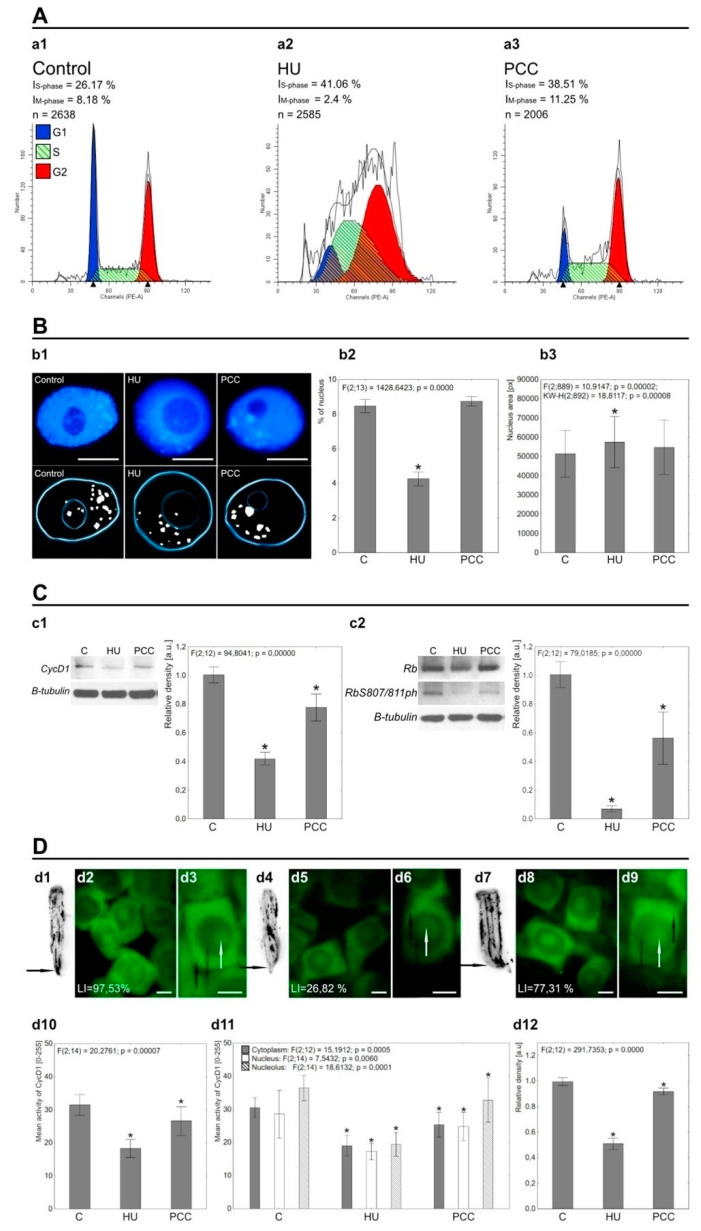
(**A**) The results of cell cycle analysis conducted using flow cytometry. The number of S-phase (I_S-phase_) cells increases significantly after HU treatment (**a2**), which is an effect of HU inhibition of the replication itself (cell cycle synchronization; S-phase arrest; cell cycle checkpoint activation). At the same time, the percentage of actively dividing cells (I_M-phase_) is visibly lowered compared to control cells. Co-treatment with HU and CF (PCC, as shown at **a3**) increases both the S-phase and the mitosis percentage, which occurs due to the cell cycle checkpoint overriding caused by caffeine. (**B**) The effects of HU and HU/CF treatment on the heterochromatin regions of nucleus. The DAPI-stained images at **b1** (upper images) were thresholded (lower images) to show only the heterochromatin (white spots). The light blue lines mark the areas of the nucleus (outer lines) and nucleolus (inner lines). After induction of replication stress (**b1**, HU), large parts of heterochromatin are left unreplicated. It is well-visible as the significantly lower percent of heterochromatin area compared to the area of a nucleus. Additionally, nucleus area becomes visibly larger (compare with **b3**, HU). Induction of PCC restores the percentage of heterochromatin in the nucleus as well as nucleus area to the levels similar to control samples (**b2**, PCC and **b3**, PCC). (**C**) The results of Western blotting for CycD1 (**c1**), Rb (**c2**) and RbS807/811ph (**c2**) proteins, the β-tubulin was used as an loading control. Values for modified Rb protein (RbS807/811ph) were normalized to the internal control, the global Rb (**c2**). The activity of both CycD1 and Rb-like protein (RbS807/811ph) was significantly diminished after HU and PCC treatments compared to control. Induction of PCC, however restored some of the activity. The charts show the relative density of blot bars, where the C (control) bar is presented as the benchmark, the units are arbitrary. (**D**) Tissue printing (**d1**, **d4** and **d7**; control, HU and PCC, respectively) of *V. faba* roots showing the accumulation of CycD1. The arrows mark the root meristem areas that were subjected to protein density measurements. Images **d2**, **d5,** and **d8** show the immunocytochemical detection of CycD1 in control, HU and PCC series, respectively. Images **d3**, **d6,** and **d9** are closeup images relative to the preceding photos in terms of experimental series. The white arrows visible on the closeups mark cytoplasm areas of the cell, the empty black arrows mark the nucleus areas and the black arrows mark the nucleoli. The cells were stained with AlexaFluor 488. The **d10** chart displays the general mean activity of CycD1 within the cells and its changes after HU and PCC induction, while the **d11** chart shows the mean activity within specific cellular compartments—cytoplasm (gray bars), nucleus (white bars), and nucleolus (stripe bars). The activity is shown as the mean direct luminescence of the protein, ranging from 0 (no activity) to 255 (maximum activity). The **d12** chart displays the results of CycD1 density as measured by the tissue printing-acquired images. Significant changes in CycD1 activity are consistent with the results of Western blotting. The differences were assessed by one-way ANOVA followed by post-hoc (Tukey’s) test at the significance level of *p* = 0.05 (asterisk).The scale bar is approx. 10 µm.

The results obtained by the cell cycle analysis revealed a significant decrease in the number of cells undergoing mitotic division after incubation with HU (2.4% of HU-treated cells as compared to 8.18% of control cells; Figure 1a1,a2, respectively) and an increased population of S-phase cells (41.06% after HU as compared to 26.17% in control; Figure 1a1,a2, respectively). The induction of PCC (co-treatment with HU and CF; Figure 1a3) triggered a vivid response in treated cells, leveling up both M- and S-phase indices to values higher than the control (11.25% of M-phase cells and 38.51% of S-phase cells after PCC). Heterochromatin areas were also notably altered after HU treatment, as seen in Figure 1B. The thresholded images (Figure 1b1, lower row) revealed that despite no noteworthy change in the quantity, heterochromatin (HC) clusters were visibly smaller when compared to the control cells and thus occupied less space in the nucleus (Figure 1b2). Additionally, HU treatment caused the chromatin to expand, which resulted in a larger nucleus area (compare with Figure 1b3). The induction of PCC, however, seems to reverse these changes back to a state that is very similar to the control as there were no statistically significant changes between the percentage of nucleus area occupied by HC clusters or the area of the nucleus itself as compared to control.

**Figure 2:**
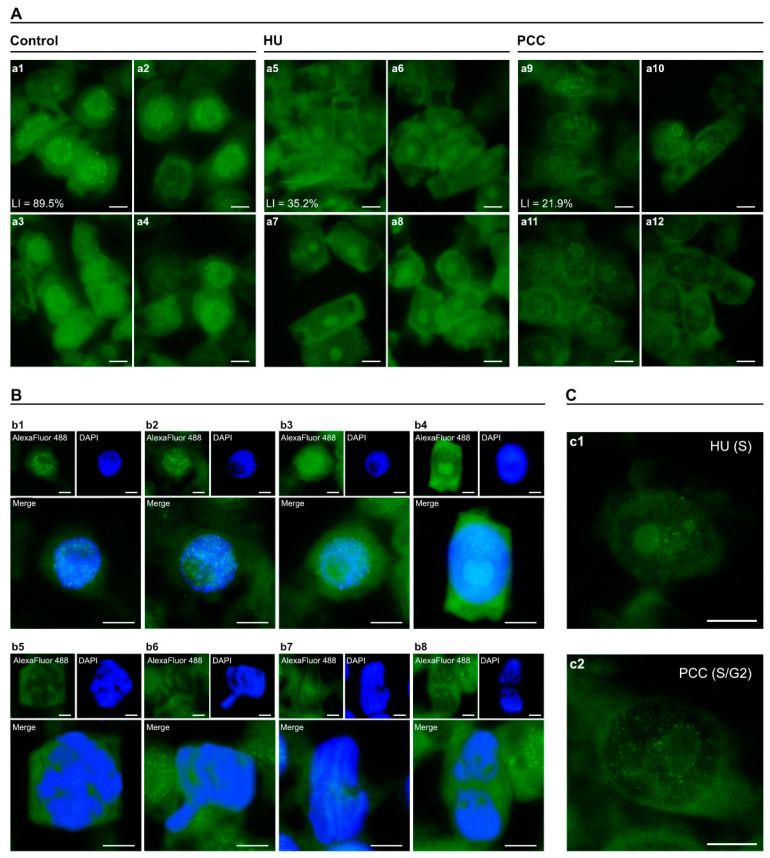
(**A**) The visible changes in activity of RbS807/811ph in interphase cells from control (**a1**–**a4**), HU-treated (**a5**–**a8**), and PCC-treated (**a9**–**a12**) series. The protein foci are significantly less visible after incubation with HU, but the pRb’s activity is moderately increased in PCC-type cells, which suggests the reactivation of Rb phosphorylation. LI stands for Labeling Index. (**B**)Representative images of cell cycle progression: **b1**—G1-phase; **b2**—early S-phase; **b3**—late S-phase; **b4**—G2-phase; **b5**—prophase; **b6**—metaphase; **b7**—anaphase; **b8**—telophase. Large images are the joined photographs of nuclei, the blue and green channels were added to each other. (**C**) The closeup images of HU-treated cells (**c1**, S-phase) and HU/CF-treated cells (**c2**, S/G2-phase), showing the differences in the number, localization, and luminescence of singular foci. The cells were stained with AlexaFluor 488 (green fluorescence) and DAPI (blue fluorescence). The scale bar is approx. 10 µm.

Western blot labeling confirmed both CycD1 and Rb, as well as RbS807/811ph present in *V. faba’s* root meristem interphase cells (Figure 1C). Densitometric analysis showed that the levels of both proteins were lowered after HU and PCC induction with the lowest being caused by hydroxyurea. Co-treatment with caffeine restored some protein activity, but the final measurements were still far from close to the control. Lower levels of CycD1 activity caused by HU were also validated by tissue printing (Figure 1d12) and by immunocytochemical detection (Figure 1d10). The changes observed in a specific cell compartment (i.e., cytoplasm, nucleus, nucleolus) occurred in a similar way. The biggest drop in CycD1 activity was observed for HU. However, CycD1 labeling remained consistently equalized. On the other hand, the pRb activity profile can be described as unequal with a large number of usually small foci (compare with Figure 2 Control).

The levels of pRb activity were also altered by HU and PCC, but in a slightly different manner. The loss of activity after incubation with hydroxyurea included the whole nucleus area, but the patterns of most active foci seem to be maintained (compare Figure 2 HU and Supplementary Figure S1a1). Induction of PCC, again, did not cause a partial reactivation of Rb-like phosphorylation, on the contrary, pRb levels after PCC were even lower than after HU treatment (Supplementary Figure S1a1,a3). Even though there were a small number of cells unaffected by HU or PCC, the overall intensity of general nuclear activity and the activity of singular foci visibly lessened in HU and were even lower in PCC cells (Supplementary Figure S1B,C).

Retinoblastoma protein activity was the most prominent interphase and diminished during mitotic division (compare with Figure 2B). As explained earlier, the forthcoming results focus on interphase cells exclusively, especially on the changes in the patterns of foci localization (compare with Supplementary Figure S1C).

### 2.2. Nucleus and Foci Analysis Leading to the Identification of Marking Types

**Figure 3:**
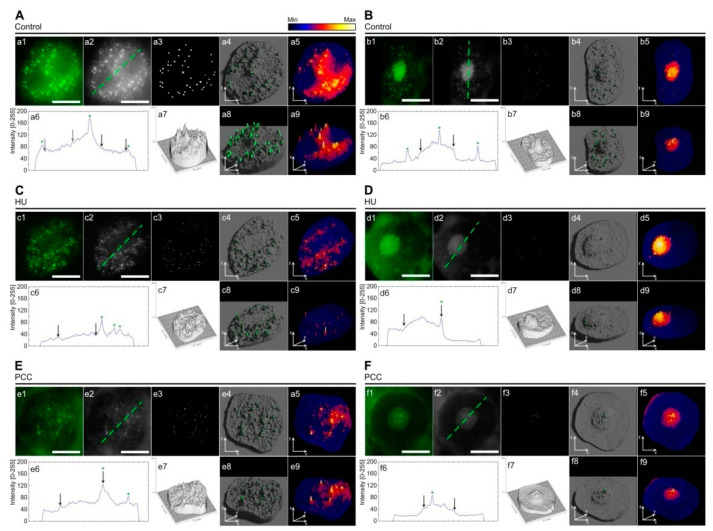
The descriptive analysis of different pRb foci localization depending on the visual morphology of nucleolus. The pRb activity within nuclei containing ‘dark’ nucleolus (**A**,**C**,**E**) is focused mostly in the other chromatin areas outside the nucleolus while the cells with ‘light’ nucleoli (**B**,**D**,**F**) show the activity of pRb also within the nucleoli itself or on the very border between the nucleolus and the rest of the nucleus (the perinucleolar heterochromatin). The green dashed lines at the grayscale images (**a2**, **b2**, **c2**, **d2**, **e2,** and **f2**) mark the pathway used to prepare the line plots (**a6**, **b6**, **c6**, **d6**, **e6,** and **f6**) that show the intensity (activity) of pRb at any given point along the line. The activity is shown in the same manner as described in Figure 1 (from 0 to 255), though the measurements are direct and not mean. The black arrows point to the nucleolus/nucleus borders, the green stars show the peaks corresponding to an active foci. The binary images (**a3**, **b3**, **c3**, **d3**, **e3,** and **f3**) display only the most active areas of pRb within the nucleus. Images **a7**, **b7**, **c7**, **d7**, **e7,** and **f7** are linear heightmaps generated in *ImageJ* based on the activity of the protein, where higher activity is shown as a peak or rise in height. The fourth column shows the 3D models based on the original images of analyzed nuclei. The grayscale activity images were used to generate the height-based mesh of the nucleus and the binary images were used to show only the active pRb foci. The last column shows the 3D models displaying the general luminescence of the analyzed nuclei in the form of heatmap, which uses the flame—like gradient to show the levels of lightness. The gradient ranges from black (no luminescence) through blue, purple, red, orange (increasing luminescence) ending at yellow and white (maximum luminescence). Both models allow to easily compare the general level of activity (5th column) with the localization of specific pRb foci (4th column). 3D models are shown as a top view (images 4 and 5 in each part from A to F) and rotated by 45° along X axis (according to the software’s coordinates). 3D models were generated in Blender 2.9.1. and based on original, unedited data. The scale bar is approx. 10 µm.

General foci localization can be characterized with respect to the fluorescent intensity of the nucleolus (either non-fluorescent or displaying visible fluorescence), as pictured in Figure 3.

On average, cells with nuclei that have ‘dark’ nucleoli (Figure 3A,C,E) tend to have a majority of their active areas outside of the nucleoli, with a small (or null) number of foci located near the perinucleolar border. “Light”-nucleoli nuclei, on the other hand, either only have a number of pRb foci within the nucleolus area or scattered evenly across the nucleus, including the nucleolus (Figure 3B,D,F). Under normal conditions, general pRb activity in nuclei with light nucleoli is visibly lower than for those which are dark—as is clearly shown by the line plots (compare Figure 3a6,b6). The y axis range (intensity) is the same for every sample analyzed in Figure 3. The higher-lower nucleus activity with regard to the nucleolus is preserved in PCC-induced cells (Figure 3E,F), but vanishes after HU treatment (Figure 3C,D), resulting in nuclei characterized by similar ranges of pRb activity. This relationship was visible in 3D heatmap models presented in Figure 3 (last column, images with the numbers 5 and 9). The blue color indicates that a marginal amount of green fluorescence was detected, which is basically the auto-fluorescence of the nucleus and thus does not represent the activity of pRb-like protein. Retinoblastoma protein activity was visible in the areas with colors ranging from red, through orange, to yellow and white (with activity levels ascending, respectively). The most active pRb foci can sometimes be found in the nuclei that show a low overall activity—an example of this is Figure 3B—the control cell with a light nucleolus and dark nucleus. The most active foci were distributed regardless of general activity, as one can see by comparing the 3D heatmap with the 3D foci activity model (Figure 3. 4th column, pictures with the numbers 4 and 8). 3D heatmaps also confirm previous data that there was a vast drop in pRb activity after HU and a moderate restoration after PCC. Interestingly, however, the light nucleolus cells after HU treatment have the most active nucleolus area, even when compared to the control.

**Figure 4:**
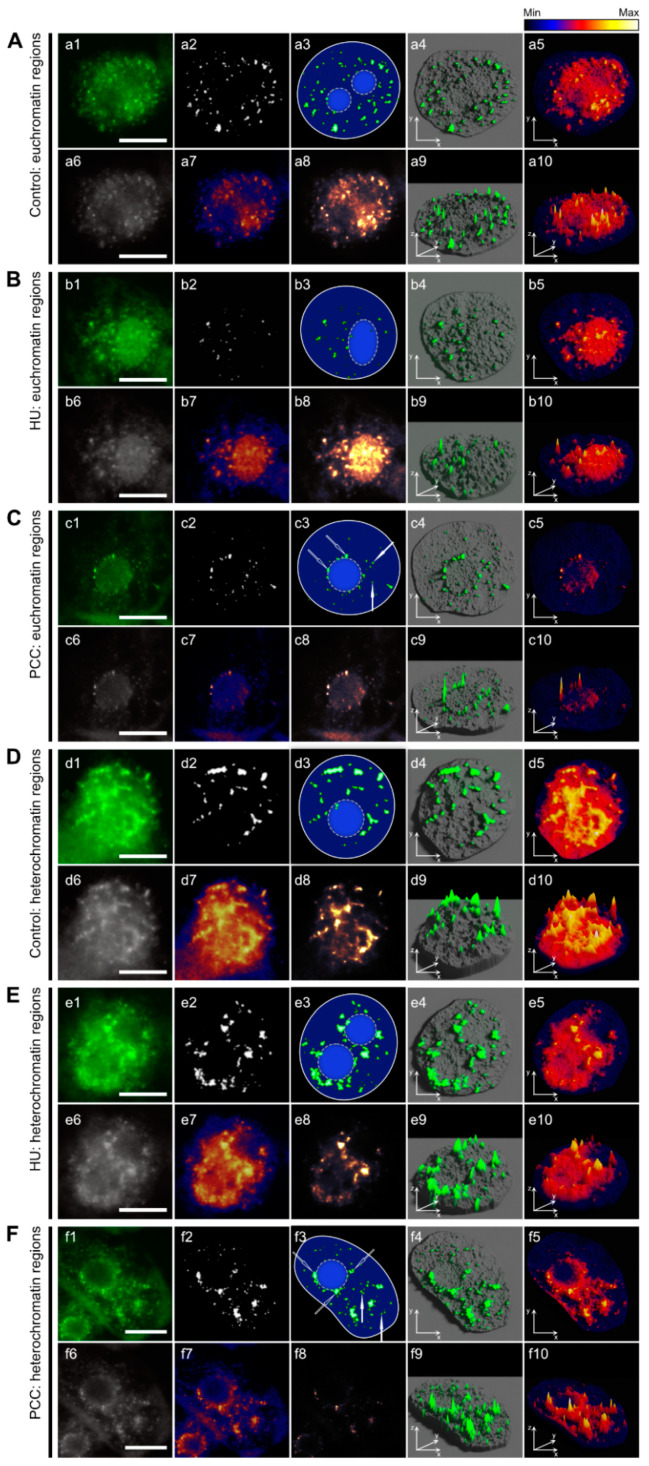
The differences between the pRb activity in euchromatic (**A**–**C**) and heterochromatic (**D**–**F**) regions of the nuclei were compared between the control, HU, and PCC series. Images with number 1 are original images acquired via fluorescence microscope (AlexaFluor 488). Images with number 2 are binary images, thresholded to show only the most active pRb foci. Images with number 3 are the drawings representing the localization of pRb foci with regard to the nucleus/nucleolus regions. Images with the number 6 are grayscale images obtained directly from the originals. Images with number 7 are heatmaps displaying the levels of luminescence, prepared as described in Supplementary Figure S1. Images with numbers 4,5,9, and 10 (4th and 5th column) are 3D models prepared also as described in Supplementary Figure S1. Euchromatin regions are usually characterized mostly by a large number of relatively small foci of pRb, while foci located at HC regions are clustered and thus form larger areas of pRb activity. The most characteristic change occurs in HC areas after PCC induction (**F**), where singular pRb foci remain clearly distinguishable, even though they are still clustered, resulting in an in-between visual pattern. The most active areas are the perinucleolar regions (shown at **f3** with white hollow arrows) where the pRb foci form a clearly recognizable ring. The white arrows show the most active regions outside of the perinucleolar chromatin. The scale bar is approx. 10 µm.

Some visible differences in pRb activity can also be observed with regards to the euchromatin and heterochromatin regions, as presented in Figure 4. Retinoblastoma protein activity within the EC regions (Figure 4A–C) tends to follow the pattern of small, singular foci while HC-related pRb forms larger clusters (Figure 4D,E,F), which are well-visible in binary images (Figure 4. Images with the number 2) as well as in the 3D foci activity models (Figure 4. 4th column, images with the numbers 4 and 9). Hydroxyurea treatment lowers the number of active foci in euchromatin (EC) regions (Figure 4B) as well as their size (compare with Supplementary Figure S2a1,a2). Although PCC induction also lowers the quantity of pRb foci (Figure 4C), their average size seems to revert back to values close to control (Supplementary Figure S2a1,a2). This is true to some extent for both EC and HC regions, however, our team also observed a very large diversification of foci size after PCC, which was shown in the form of surprisingly high values of standard deviation (Supplementary Figure S2a2). Heterochromatin- (HC-) related clusters of pRb in control cells seem to “flow” into each other (Figure 4D). Induction of replication arrest or PCC tends to accentuate singular clusters (HU) or singular foci that were grouped together, usually without the “flowing” effect (PCC). This partial loss in the clustering effect was consistent in every observed nucleus.

The repeat occurrence of pRb foci at the border between nucleus and nucleolus showing up in PCC-type cells was also noticeable (Figure 2c2; Figure 3E; Figure 4C,F). This feature was observed within each experimental series and will be discussed further in this article.

The positive correlation between the number of active foci and their total area (Supplementary Figure S2B) was anticipated. According to our findings, cells treated with HU still display similar correlation coefficients, despite lower pRb activity. Co-treatment with caffeine, on the other hand, introduces a new fraction of nuclei with very few but larger than normal foci (Supplementary Figure S2b3,c3, marked with a red dash rectangle). We have found similar dependencies between the number of foci and their average size (Supplementary Figure S2C), percent of nucleus area covered (Supplementary Figure S2D), and between the average foci size and percentage of nucleus area (Supplementary Figure S2E). Each time, the degree of correlation in the HU series was close to the control. Cells with PCC symptoms, however, always displayed some anomalies. This lead to the conclusion that even under replication arrest, nuclear stress response mechanisms still work properly as opposed to PCC. The scatterplots shown in Supplementary Figure S2B–E are composed of raw data, lower numbers of HU inputs result from a very low pRb labeling index in the cells treated with hydroxyurea (35.2% in HU, 89.5% in control; one should refer to Figure 2A).

### 2.3. Retinoblastoma ProteinHas Five Labeling Profiles in Interphase Cells

**Figure 5:**
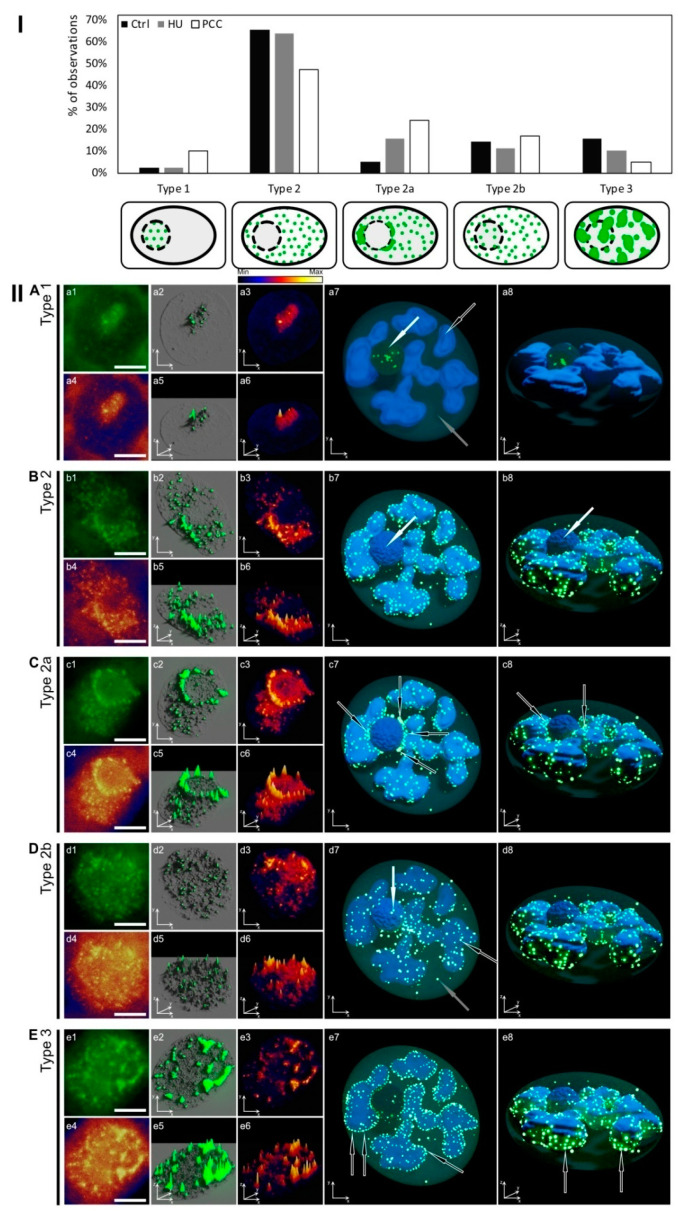
(**I**)The schematic characterization and percentage of observed cells displaying five different types of pRb activity patterns in normal conditions. Types 1, 2, 2a, and 2b are generally characterized by relatively small pRb foci, while Type 3 is specified by foci clustered into larger compounds. Type 1 displays a small number of pRb foci located only within the area of the nucleolus. Type 2 includes small active foci in the area of the nucleus, without the activity in the nucleolus. Type 2a is very similar to Type 2 with the addition of a higher number of active pRb foci (often formed into little clusters) in the perinucleolar heterochromatin regions thus forming a very unique ring around nucleolus. Type 2b shows pRb activity scattered evenly on the whole nucleus area, including the nucleolus. Type 3 shares the characteristics of Type 2b with the difference in the size of the foci, which here form visibly large clusters. (**II**)The original images and their modifications represent all five types of pRb activity profiles as described earlier in Figure 5I. Images with number 1 show the unedited photographs obtained from fluorescence microscope, stained with AlexaFluor 488. The images with number 4 show the intensity of fluorescence displayed as a heatmap (as explained in Figure 3). Images with numbers 2 and 5 show the 3D models based on the original images with only the most active foci marked as green glowing parts, and images with numbers 3 and 6 display the 3D models with the general nucleus activity showed as a heatmap (as also explained earlier in Figure 3). The images with numbers 7 and 8 are the general 3D models developed according to the conclusions derived after analyzing individual types of pRb activity. The white arrows mark the nucleolus, the white-framed black arrows mark the regions of intensified pRb activity in heterochromatin areas (heterochromatin areas are presented as light blue shapes), and the gray arrows mark the euchromatin. The scale bar is approx. 10 µm.

We were able to distinguish five pRb labeling profiles based on the size and localization of foci within the nucleus. The schematics describing each profile and the percentage of cells showing the specific Type are shown in Figure 5I. The criteria that determined the following Types were as follow: (i) Type 1—small pRb foci located only within the nucleolus (*no*) area; (ii) Type 2—small foci distributed evenly outside of the nucleolus (*no* is not labeled); (iii) Type 2a—the same as Type 2 plus a number of highly active pRb foci forming a ring on the perimeter of the nucleus/nucleolus; (iv) Type 2b—small foci distributed across the entire nucleus (including nucleolus) and (v) Type 3—large clusters of pRb covering the entire nucleus (including nucleolus).

The in-depth visual analysis of particular labeling types in control cells is presented in Figure 5II. The general 3D models (Figure 5II images with numbers 7 and 8) were used for high-res visualizations of activity. The ranges in the x and y axes were equalized where possible in Supplementary Figure S3 for easier comparison of particular variables between Types and series.

All nuclei displaying the Type 1 profile have a light nucleolus region (clearly visible in Figure 5IIa1,a3,a4,a6). Retinoblastoma protein activity, however, was visibly lower compared to other Types (compared with Supplementary Figure S3a1). The number of active foci was also understandably lower (Supplementary Figure S3a2,a4,a5), but they were slightly larger than the foci of Types 2, 2a (only in those furthest from the perinucleolar ring), and 2b. There was a moderate correlation between the size of particular foci and the level of pRb activity in this region (Supplementary Figure S3b1), although size did not appear to be correlated with quantity (Supplementary Figure S3d1). Type 1 labeling is least commonly observed.

The most common Type 2 labeling was composed of a rather large number of small-sized foci. Clustering may occur, but this feature is not really prominent. The most characteristic feature was a usually dark nucleolus with no pRb activity visible (compare with Figure 5IIB). The foci size was almost uncorrelated with pRb activity (Supplementary Figure S3B,b2), which means that the general activity within the nucleus depends on the sole number of active spots rather than the brightness of a specific foci.

The most characteristic feature of Type 2a was the perinucleolar ring of highly active pRb foci that appears to be “added” to Type 2 labeling (Figure 5IIC, marked with arrows on c7 and c8). The ring shows much more pRb activity than the rest of the nucleus as seen on the heatmap models (Figure 5IIc3,c6), and has the strongest fluorescence intensity when compared to other Types (Supplementary Figure S3a1). This Type is also characterized by the largest number of active foci (Supplementary Figure S3a2). Interestingly, the increasing number of foci in Type 2a nuclei correlates with an increase in their size (Supplementary Figure S3d3), which seems to be quite uncommon in comparison to other types.

The Type 2b activity profile is also composed of a large number of small pRb foci (Figure 5IID), however this time, both nucleus and nucleolus regions are labeled. The nucleolus is either dark and not visible after immunocytochemical staining (as in Figure 5IId1) or light and clearly recognizable (as in Figure 3B). Foci number, size, and nucleus coverage are comparable with the characteristics of Type 2 (Supplementary Figure 3a2–a5), but the overall pRb fluorescence is statistically lower, mostly because of the “light nucleolus” nuclei’s low activity (as explained earlier). This type also shares the same lack of correlation between the size and intensity of the foci as Type 2 (compare Supplementary Figure S3b2,b4).

The Type 3 profile stands out from others because of the size of pRb active areas, which group together and often merge into clusters (Figure 5IIE). Small-size foci (as in other types) are also present, but their population is relatively small. Though the clusters usually appear as if they have melded together, there are still some extremely active singular foci visible within the clusters (Figure 5IIe3). The nucleolus here is “dark” and not distinguishable after immunocytochemical staining. This type has the largest area coverage of the nucleus by pRb activity (Supplementary Figure S3a4,a5). Interestingly, the number of foci is also the highest (Supplementary Figure S3a2) and this Type shows very little correlation between foci number and size (Supplementary Figure S3d5).

### 2.4. Retinoblastoma Protein Patterns Alterations Caused by HU and PCC

Although pRb activity is significantly altered in response to replication arrest caused by HU or cell cycle override caused by PCC, the patterns of labeling are still clearly identifiable. Observed differences occur mostly in terms of (i) the number of observed cells and (ii) some specific areas of higher/lower local activity, but the patterns themselves are not really disturbed. Due to the loss of pRb activity and thus the lower number of foci within the nuclei, the patterns are also less “dense”.

The most visible alteration is related to the changes in the populations of cells displaying a particular labeling pattern. Type 2 is the most common Type in every series, but the number of Type 2 cells is visibly lower after PCC induction (Figure 5I). At the same time, the number of observations of Type 1 cells is the same for control and HU, but increases sharply after PCC meaning there are more cells with activity within the nucleolus. Type 2b follows changes similar to Type 1 (Figure 5I). The most striking difference occurs with Type 2a cells whose numbers change from under 10% in control through to 15% in HU and at over 20% in PCC series. The population of Type 3 cells is lower after HU treatment and lowest after PCC induction (Figure 5I). A common factor that one should note is that after the induction of replication stress and, to a larger extent, PCC, the fraction of cells with nucleolus-related pRb activity rises.

Another interesting observation comes from the analysis of the changes in pRb activity with regards to the specific pattern (Supplementary Figure S3E) and percentage of nucleus covered by pRb (Supplementary Figure S3F). The mean amount of foci activity in all experimental series (not divided into labeling types) was presented earlier, at Supplementary Figure S1a3, and showed a significant loss in fluorescence after HU treatment and an even higher loss after PCC induction. At first glance, there is not much of a difference between changes in the activity of labeled types. A closer look, however, reveals some subtle details. Type 1 does not actually show any significant alterations in its activity under stressful conditions (Supplementary Figure S3e1). Type 2a, despite being significantly different from control, has levels of activity similar to HU. Type 3 does not exhibit any visible changes after HU treatment compared to control. The percentage of the nucleus area covered by pRb foci for whole-cell populations is presented in Supplementary Figure S2a4. According to this general analysis, replication arrest causes a massive drop in the area of the nucleus labeled by pRb. The induction of PCC restores some of the active sites, yet the percentage is still significantly low. Type 1, again, shows no real changes of this variable between the experimental series (Supplementary Figure S3f1). Type 3 shows a change in its pattern similar to earlier results (in Supplementary Figure S3e5) there is no real difference between HU and control cells, but after PCC induction, the percentage of nucleus area is greatly diminished (Supplementary Figure S3f5).

**Figure 6:**
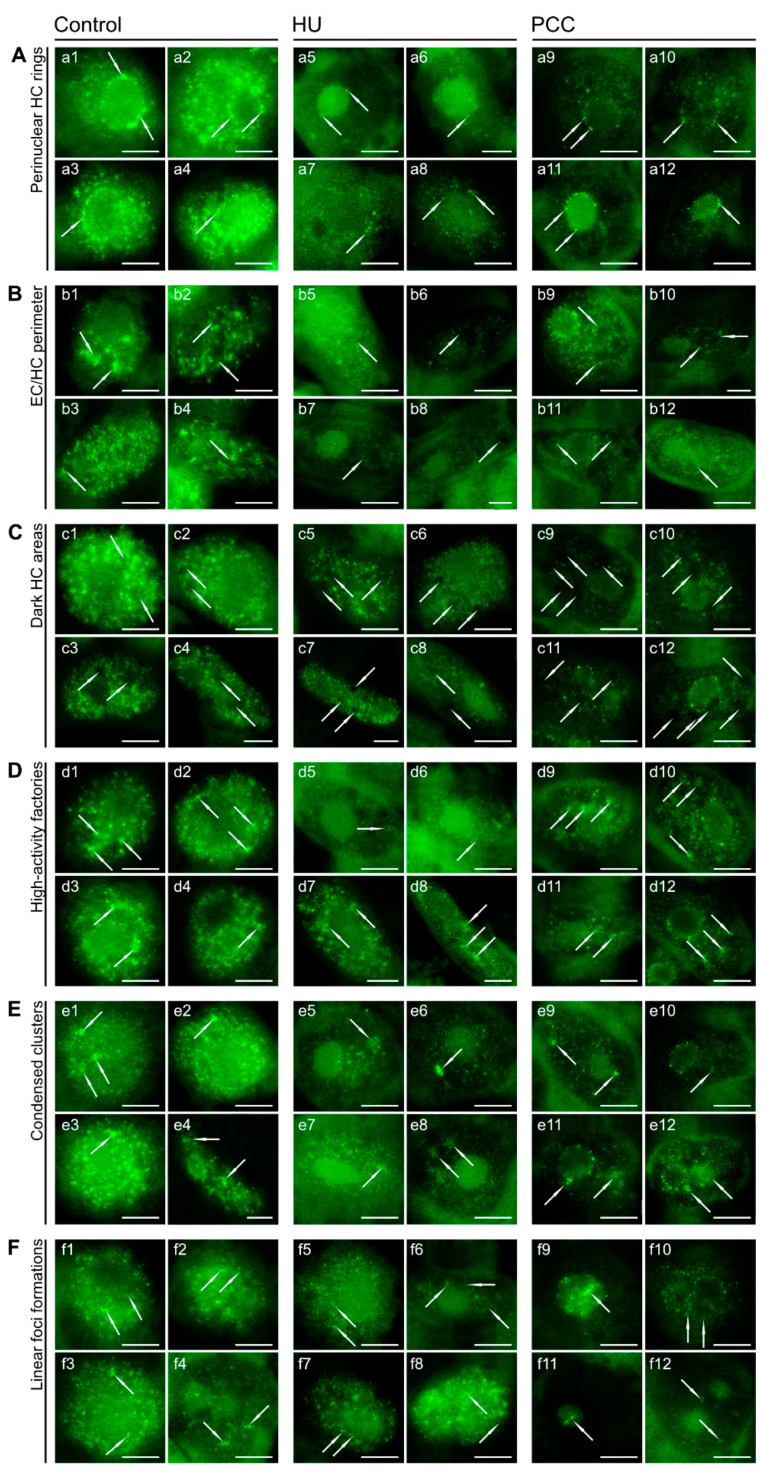
The differences between various characteristic pRb activity details (all marked with white arrows) under normal conditions, after induction of replication stress and PCC. (**A**) The perinucleolar rings that are very easily recognizable in control cells are usually composed of a large number of small foci that tend to form into minor clusters. Due to the lowered number of active foci in HU and PCC series, the clustering effect is not visible. (**B**) The perimeters of EC/HC regions tend to have the second strongest (after the perinucleolar rings) grouping of pRb foci, resulting in cluster formations. (**C**) The heterochromatin areas that lack pRb activity are much more prominent after HU or PCC induction as compared to the control. (**D**) The high-activity factories are composed of a high number of small and grouped together, yet not clustered pRb foci. They are less visible after incubation with HU, but their levels of activity return close to normal after PCC induction. Due to the fact that the rest of nuclear activity after PCC is still lower than control, they are much more distinctive. (**E**) Highly condensed clusters are relatively small areas containing a large number of very active foci. Even though HU treatment induces a decrease in general pRb activity, those clusters are still significantly active even under replication stress or after PCC. (**F**) Linear foci formations are very peculiar arrangements of a small number of pRb foci, forming nearly straight lines and displaying the tendency to be clustered (visible as a light ‘halo’ connecting all lined up foci). They seem to appear mostly outside of the nucleolus in control and HU cells (**f1**–**f8**), but induction of PCC displays their occurrence also within nucleoli (**f9** and **f11**). The cells were stained with AlexaFluor 488. The scale bar is approx. 10 µm.

Some of the peculiar, local pRb foci formations that are present in control are also usually slightly altered after HU or PCC. The perinucleolar rings, unique to Type 2a labeling (Figure 6A) are far less visible after HU treatment, largely due to the loss in the quantity of foci. There are usually a couple of single foci (two or three), and the clustering effect is generally nonexistent (Figure 6a5,a6). On occasion, the “rings” seem to wander further from the nucleolus border into the nucleus but still retain the ring shape (Figure 6a7,a8). Induction of PCC restores some of the foci quantity, resulting in more prominent rings (Figure 6a9,a10), but the clustering effect is still minimal as the foci are quite small and rather condensed (Figure 6a11,a12).

The EC/HC perimeter (Figure 6B) is usually the area with the highest activity of pRb, which is quite characteristic for Type 3 labeling (Figure 6b1). It usually follows the same silencing pattern as HU and PCC, but after co-treatment with caffeine, the areas of high/low activity are much more visible, unlike for HU (compare Figure 6b7,b11, for instance). Overall, the dark areas of pRb-inactive chromatin (Figure 6C) tend to have depleted levels of fluorescence (Figure 6c5–c12).

On the other hand, some high-activity regions (Figure 6D), although less prominent after HU (Figure 6d5,d6), are still clearly visible, and the cells that were only slightly affected by replication arrest display these regions similarly to control. PCC induction, again, causes an increase in the contrast between the high-activity regions and the rest of the labeled nucleus (Figure 6d9–d12).

The condensed clusters (Figure 6E) should not be mistaken with the clusters which are characteristic of the Type 3 activity profile. The clusters presented here are rather small and composed of a number of singular foci, grouped together yet not “merged” into one big drop. The clustering effect occurs, but it is minor, and every unique foci are still visible. These elements tend to still be quite active (although the general activity is lower) after HU and PCC induction, which is clearly visible in Figure 6e5–e12.

The smallest and most unique are the rare, linear foci formations (Figure 6F) composed of 2-5 singular foci, lined up almost perfectly and exhibiting a slight clustering effect. These structures are more prominent in HU and PCC series. PCC-induced linear formations tend to be bigger (Figure 6f9) and more frequent.

### 2.5. Histones

**Figure 7:**
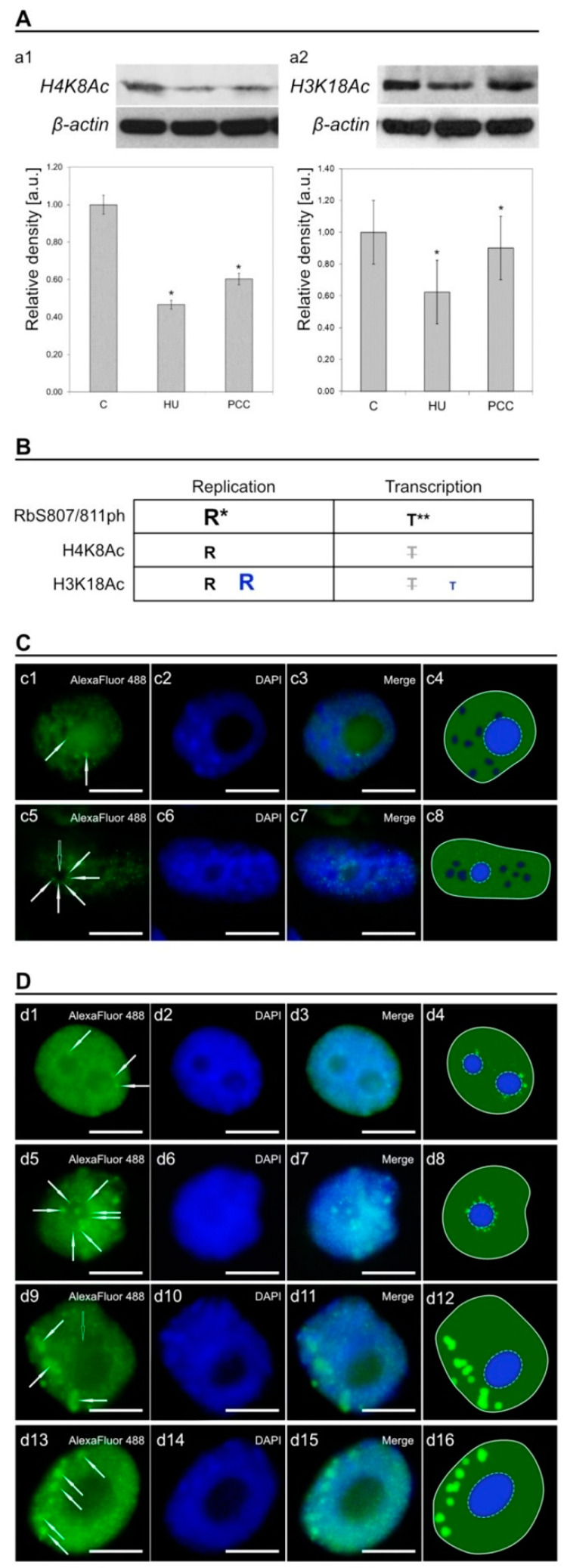
(**A**) The results of Western blotting for H4K8Ac (**a1**) and H3K18Ac (**a2**). β-actin was used as the labeling control for both histones. The density of bars is presented on the charts in arbitrary units, the control group is used as a benchmark. Stars show the significant differences between control cells and the given analyzed group. (**B**) The table summarizing the conclusions between this research (represented by black letters) and the research conducted by Jasencakova [52] (represented by blue letters). R stands for replication, T stands for transcription. The bigger the letter is, the more the protein modification (phosphorylation for Rb-like or acetylation for histones) is connected with a given mechanism (replication or transcription). Semi-transparent and crossed-out letters indicate that the connection was not analyzed. R* and T** mean that phosphorylation of Rb-like protein appears to be linked mostly with the replication (R*), but some small fraction of its active foci could possibly be related to the transcription (T**) because of the high colocalization of pRb foci on the perinucleolar regions in some instances. (**C**) Immunocytochemical detection of H4K8Ac. (**D**) Immunocytochemical detection of H3K18Ac. The cells (both in **C** and **D**) were double-stained with AlexaFluor 488 (1st column) and DAPI (2nd column). Images in the 3rd column are merged images of both fluorophores. Images in the 4th column are schematics representing histone localization in the nucleus. The light-blue ellipses with dashed lines represent nucleoli, the green color represents the histone proteins, and the blue color—chromatin. The white hollow arrows at c5 and d9 mark a large heterochromatin area lacking histone activity. The white arrows mark the singular areas of higher histone activity. The scale bar is approx. 10 µm.

A comparison of the activity of histones H4K8Ac and H3K18Ac was performed as an aid to determine the role of pRb activity in *V. faba’s* cells during replication arrest and after cell cycle checkpoint bypass. Western blotting analysis showed a significant decrease in the amount of both histones (Figure 7A) with a pattern indicative of the changes in pRb (compare with Figure 1c2). The table in Figure 7B is a summarized conclusion of the possible relation between pRb activity, replication and/or transcription in HU and PCC induced cells. Our team concluded that most of pRb activity was actually related to DNA replication. There was, however, a subpopulation of pRb foci in the nucleus that were most probably transcription-related (which will be discussed in the next part of the article). After analysis of the activity based on immunocytochemical detection, we observed some similarities in chromatin labeling between pRb, H3, and H4 histones, as shown in Figure 7C,D, marked with white arrows.

## 3. Discussion

### 3.1. Prolonged Hydroxyurea Incubation Causes Heterochromatin Under-replication, Suppresses Mitotic Divisions, and Diminishes CycD1 and RbS807/811 Activity

Hydroxyurea is a factor well-known for arresting cells in the G1/S phase and blocking replication due to its inhibition of the enzyme ribonucleotide reductase (RNR), which results in the depletion of dNTP pools [53]. The flow cytometry results confirm that the same effect is observed in *Vicia faba’s* root meristem cells (Figure 1A), as one can see a significant increase in the number of S-phase cells, most of which were arrested in the early-S-phase. Once this has taken place, stalled replication forks require the ATR-dependent mechanisms to react and prevent them from collapsing even if the replisome components usually disengage from the DNA [54]. Induction of PCC “shifts” the cell cycle by bypassing the ATM/ATR-dependent checkpoint, forcing the cells to progress into the mitotic division regardless of whether the replication is finished. Our previous studies have shown that PCC-type cells are prone to start mitosis with heavily under-replicated chromatin causing vast chromosome aberrations. This was not observed in cells that were arrested in S-phase [2,4]. Heterochromatin areas seem to be of the highest importance in terms of a cell’s response to replication arrest as studies show that HU frequently causes the accumulation of ssDNA and leads to epigenetic alterations within these regions [50,55]. Replication stress has been proved to facilitate the expansion of heterochromatin regions at multiple loci (compare with Figure 1B), possibly by the methylation of H3K9 [56] and silencing of heterochromatin-associated PEV [55], among others. The HU-induced spreading of chromatin seems to be more prominent the longer the treatment abides and seems to be locus-specific, affecting the constitutive and facultative heterochromatin areas primarily [57]. These structural changes may be inherited epigenetically, and previous studies show that this phenomenon is evolutionarily conserved [57]. It is interesting to note that PCC induction reverses heterochromatin expansion of HU-treated cells back to ‘normal’ levels even in interphase cells (compare Figure 1B). Little is known, however, about the underlying mechanisms that drive this reversal in cells exposed to the inhibition of ATM/ATR kinases. We theorize that it may involve cohesin activity and nucleosome modification mechanisms responsible for chromatin compaction during the quiescence, but more detailed research into this matter is required. Other studies have shown that cohesins contribute greatly to the primary chromatin structure, and their absence alters the residence of nucleosomes in some regions [58]. Additionally, transcription-dependent chromatin remodeling may also be caused by the tumor-suppressing protein pRb which is involved in the nucleosome remodeling and histone modifications (e.g., acetylation/deacetylation or methylation) [13,17,40].

The aberrant activation of Rb-E2F in cancer-developing cells is usually an effect of reduced levels of dNTPs [59], which was replicated in this research with the use of HU. The canonical mitogenic pathway of G1/S transition involves CycD-activated CDK4/6 complexes, which lead to the phosphorylation of pRb and, in turn, the release of E2F, previously repressed by hypophosphorylated pRb [34,35,59]. We decided to take a preliminary look at both CycD1 and RbS807/811ph in *V. faba* cells. Our data show that prolonged HU treatment causes a drop in the activity levels of both proteins, and these levels are slightly restored after the induction of PCC, but not to their original extent (Figure 1C). As a matter of fact, a decrease in activity was the only notable change in CycD1 (Figure 1D). pRb also displayed some prominent changes in the patterns of activity regarding its location in the nucleus (compare Figure 2A,C). This preliminary research pointed us to the initial conclusion that further analysis of interphase pRb activity profiles may shed some light on the *V. faba* cell’s response to replication arrest and the induction of PCC.

### 3.2. Retinoblastoma ProteinActivity Profiles Do Not Change Despite Induction of Replication Stress and PCC

The characteristic S-phase activity profiles of pRb distinguished in this research (Figure 5I) are—in our opinion—linked to the transcriptional needs of the nucleus at a given stage of the replication. All profiles, except Type 1 (expressing pRb activity only within the nucleolus, compare Figure 5IIA), correspond to the rates of replication during the early/late S-phase periods as analyzed by Li et al. [57], although we theorize that Type 1 may just be the very early, possibly even initial stage of G1/S transition. Stochastic events, such as replication origin firing, are usually very hard to describe based on observation. Hence, various mathematical models have to be used to help explain the empirical data. Replication dynamics depend greatly on this stochasticity and any disturbance, a stalled fork, for instance, may jeopardize the whole process [48,60,61].Replication is also an orderly advancing process, and in eukaryotes, euchromatin is generally replicated early in S-phase while heterochromatin is replicated later (with some exceptions). This scheme is conserved within the entire eukaryotic kingdom [62]. The small pRb foci observed in Types 2, 2a, and 2b pertain to the euchromatin areas, replicated vividly during early S-phase, while Type 3, characterized by larger foci, is related to the heterochromatin and late S-phase replication [57]. However, this connection is indirect as pRb only facilitates the transcription of E2F-related genes and not the replication itself.

The loss of pRb activity after HU treatment appears to be a proper cell response to the event of replication stress induction, but it does not seem to be the only cause for the depletion of dNTPs. As a matter of fact, eukaryotic cells have been shown to somehow control the levels of dNTPs, never allowing them to drop to zero [53]. Replication is arrested when dNTP levels are not above a critical threshold. This mechanism keeps the minimum required level of dNTPs as a backup should the replication restart. Recent studies confirm previous theories that cells may react to HU via some sensory mechanism other than the depletion of dNTPs. Research shows that HU treatment may produce reactive oxygen species that may also act as triggers for other stress-response mechanisms [7]. Interestingly, pRb activity patterns observed after HU still remain the same as in control cells. Their morphology may be altered because there are a lower number of foci and those present are not as active, but their localization patterns follow the same five types as in control cells. In our opinion, the areas that were already being replicated when the HU treatment started might have been allowed to finish the replication, but the cells did not activate any new origins. What is striking is the fact that even prolonged HU treatment cells exposed to HU exhibited a proper stress response, lacking any significant aberrations. Although HU induces ssDNA at the sites of stalled replication forks, *V. faba* cells do not seem to accumulate an excessive number of fragile sites even after 32h of treatment. Studies show that upon replication arrest, nucleosome components disengage from DNA [54], allowing the ssDNA-RPA complex to act as a landing site for—among others—ATR kinase [63,64] and the prevention of fork collapse. Strikingly, these results were also reported for human cells after acute HU treatment, but the same cells lacked the ability to restart replication forks after prolonged HU incubation [54]. Replication in cells unable to restart replication forks usually results in the accumulation of under-replicated chromatin areas that may lead to genome instability or death of daughter cells. *Vicia faba’s* cells seem to react in a more stable manner than human cells, at least in terms of preventing fork collapse for longer periods of time, but this feature is yet to be determined precisely.

The fact that cells maintain some minimal threshold of dNTPs, even with inactivation of RNR, may explain why one can see a slight increase in pRb (and in turn replication) activity after the induction of PCC. Should the replication stress response mechanism wait for the dNTP pool to be reduced to zero, the induction of premature mitosis would not result in the reactivation of any replication activity. However, retinoblastoma protein foci are maintained following the same patterns found in control, which means they activate the same chromatin regions as they would without the cell cycle bypass. The vast chromosome aberrations that can be observed in PCC-type cells (especially mitotic chromosomes) do not seem to be derived just from a lower activity of pRb. Caffeine is an agent well-known for its inhibition of ATM and ATR kinases [65] thus—in our opinion—aberrant structures result primarily from the ATM/ATR disruption rather than from a noneffective stress response. pRb-related mechanisms seem to work efficiently even during PCC induction as the localization patterns are subjected to changes similar to those in HU-type cells.

### 3.3. Heterochromatin

Heterochromatin plays a key architectural role in eukaryotic cells. For instance, it can ensure correct chromosome segregation by influencing the proper assembly of centromeres [66] or regulation of gene expression [67]. Studies show that constitutive heterochromatin is mostly transcriptionally silent, while facultative heterochromatin is composed of regions that are preferentially silenced and form rather large compartments, for instance, Lamina-Associated Domains (LADs) located at the periphery of the nucleus and Nucleolus-Associated Domains (NADs) located around the nucleolus [68]. Both heterochromatic compartments may be functionally redundant as some studies show they may change their location from LAD to NAD (and vice versa), should the other compartment be disrupted [69]. Heterochromatin organization ensures both the stability of gene regulation but also helps to organize the large-scale genome and form specialized compartments, including the nucleolus [68,70].

The Rb protein is particularly active in the perinucleolar region in Type 2a nuclei, as shown in Figure 5IIC. Interestingly, this is the only ″early S-phase″ replicating profile with such prominent pRb activity in this heterochromatin area. Foci forming a signature ring around the nucleolus border usually tend to cluster into larger compartments (compare with Figure 5IIc2), which is characteristic of heterochromatin regions. Replication stress is especially dangerous for the late-replicating heterochromatin simply because the early-replicating regions usually have time to finish duplication (this may be the reason why cells keep some critical threshold levels of dNTP pools). One important fact that should be highlighted here is that replication arrest does not force forks to stop replicating immediately. Cells with accumulated dNTP pools were shown to continue replication regardless of RNR inhibition [53]. Cells react, however, by inhibiting new origin firing, preventing any new initiation of replication. The most common outcome, therefore, is the under-replication of late-replicating heterochromatin [71]. One can see that the number of cells expressing Type 3 activity (representing pRb activity profile in heterochromatin areas) are much less common in HU- and PCC-type cells (Figure 5I), most probably due to the fact that these areas are restricted from starting replication. What is noteworthy is that the number of cells expressing increased perinucleolar pRb activity increases after HU and PCC treatment. This indicates that perinucleolar heterochromatin may be a key factor in response to replication stress. In fact, some other areas of facultative heterochromatin may also contribute to this response as we observed an increased activity of pRb in some small areas, usually in the perimeter of eu- and heterochromatin (compare Figure 6).

Heterochromatin is usually highly condensed and formed by denser and less mobile nucleosome groups compared to euchromatin [72,73]. This feature presents a challenge for replication forks and replisome components, which are meant to replicate heterochromatin DNA. For this reason, heterochromatin areas are more prone to replication stress caused by a stalled fork generated by an environmental obstacle or genotoxic interference (like HU, for instance). During replication, nucleosomes are disrupted ahead of the progressing replisome [74] in order to unwind DNA. After replisome passes, DNA organization is restored by reassembling parental histones and the deposition of newly synthesized histones onto DNA [49]. The mechanisms guarding the fidelity of epigenetic markings passed onto new DNA strands are still quite elusive, but recent studies show that replication stress causes disturbances in the transfer of the epigenetic markings, leading to epigenetic instability [55].

### 3.4. Histones

Apart from being a ″gateway″ protein for G1/S progression, pRb also plays a significant role in the regulation of nucleosomal structures as well as chromatin compartments, partially by its interactions with HDAC1 (histone deacetylase 1) or SWI/SNF (ATP-dependent histone exchange/removal complex) proteins [31], which modulate gene transcription by modifying the structure of chromatin. Generally, histone acetylation leads to the opening of chromatin and stimulates transcription. Retinoblastoma protein can modulate the local balance between histone acetylation/deacetylation and transcription as a result [40]. The correlation between acetylation and transcriptional activity has been proven several times, as described by Jasencakova and her co-workers [52,75]. There is, however, evidence that modifications at the lysines of histones H4 and H3 are connected with replication itself rather than transcription [52]. Histone H4 acetylation has been reported to be strongest in heterochromatic regions during replication and to correlate with the incorporation of BrdU, while the H3 acetylation patterns were mostly uniform throughout the cell cycle [52,75]. The comparison of H4K8Ac and H3K18Ac histones with pRb activity confirms general acetylation of chromatin domains occur during S-phase, as shown in Figure 7C (H4K8Ac) and Figure 7D (H3K18Ac) seem to correlate mostly with replication. Some local acetylations may be related to the transcription—their percentage, however, is minimal. In our opinion, general pRb activity in interphase nuclei is also related mostly to replication. The exception to this is the nucleolus and perinucleolar region of heterochromatin that may correspond to the transcriptional activity. This idea is supported by the fact that there is an increased number of cells expressing enhanced pRb activity in these regions after replication arrest and PCC induction (Figure 5I).

## 4. Material and methods

### 4.1. Plant Material, Growth Conditions, Hydroxyurea, and Caffeine Treatment

The seeds of *Vicia faba var. minor* (Center for Seed Production, Sobiejuchy, Poland) were germinated on Petri dishes lined with a wet filter paper, in the dark, at room temperature until the seedlings were approximately 3 cm long. Selected seedlings were divided into 3 groups and incubated in (i) water (negative control; 32h total incubation time), (ii) 2.5 mM HU (S-phase synchronization/positive control; 32h total incubation time), and (iii) 2.5 mM HU for 24h, and transferred to a mixture of 2.5 mM HU and 5 mM caffeine for 8h (PCC induction; 32h total incubation time). Roots were continuously aerated in a water-bath shaker (30 rpm). The procedure and incubation times were performed as described previously by [3]**.**

### 4.2. DAPI Staining and Quantitative Heterochromatin Measurements

The nuclei were isolated using Van’t Hoff’s method as first described in [76]. *Vicia faba’s* roots were fixated in PBS buffer (4% paraformaldehyde, pH 7.2) for 10 min at room temperature. Root apical fragments were cut off after fixation and washed in PBS buffer twice (5 min each). Lastly, they were suspended in a drop of PBS buffer and squeezed between 2 microscope slides. The nuclei released into the drop were collected and purified in a centrifuge (5 min, 600 g). Isolated nuclei, suspended in fresh PBS, were stained with a DAPI (Sigma-Aldrich, Saint Quentin, France) solution at 1 µg/mL, in the dark for 5 min. The nuclei were centrifuged after staining (5 min, 500 g at 4 °C) in order to remove the staining agent, suspended in a fresh PBS, and applied on polylysinated slides. Samples were analyzed using Nikon ECLIPSE E600W (Nikon, Warsaw, Poland) fluorescent microscope, and obtained images were used for heterochromatin measurements.

Unedited images were converted into grayscale, and chromatin density was measured as the level of each pixel’s lightness, from 0 (no chromatin present) to 255 (highly condensed chromatin). In the first step, the nuclei were marked, and the areas of each nucleus were measured as a total number of px on the image, in the second step, only the heterochromatin areas of each nucleus were marked and their area was measured (also as the number of pixels). The overall heterochromatin percentage in the nucleus was calculated as the mean heterochromatin area against the mean area of the nucleus, in percentage terms as described in [8].

### 4.3. Cell Cycle Analysis

Cell cycle analysis was performed using the RSLII flow cytometer and FlowJo 10.4.1. software (FlowJo LLC, Ashland, OR, United States), similarly to the procedure described in [64]. The forward and side scatter channels (FSC and SSC, respectively) were used for the identification of cells in different phases of the cell cycle. First, the debris was removed by analysis of SSC-A versus FSC-A plot. Next, the FSC-H versus FSC-A plot was used to remove clumps and doublets and SSC-H versus SSC-A plot to remove debris remains and some apoptotic cells. Finally, gated cells were applied to propidium iodide (PE-A vs PE-W), and the final plot was generated using cell count versus PE-A. The control sample of cells was used to validate the gating, and the same parameters were used for the rest of the samples. Final identification of the subpopulation of cells in different phases of the cell cycle was performed in FlowJo by fitting Gaussian curves to each phase.

### 4.4. Western Blotting

Approximately 1.5 mm-long root meristem sections (*n* = 30 for each series) were cut off and used for protein extraction. Analyzed proteins were extracted in accordance with [2], using TriPure Isolation Reagent (Roche Diagnostics Corporation, Indianapolis, IN, United States) in accordance with manufacturer’s instructions, and final concentrations of cell lysates were assessed by Ultrospec 110 pro (Amersham Biosciences, Vienna, Austria). Protein extracts were separated on 7% polyacrylamide-SDS gel and extracted onto a nitrocellulose membrane (u 0.45 µm, Schleicher and Schüel, Dassel, Germany). Signal visualization was performed using NBT/BCIP (Nitro blue tetrazolium chloride/5-bromo-4-chloro-3-indolyl phosphate, toluidine salt, Sigma-Aldrich, Saint Quentin, France) as substrates. β-tubulin was used as an internal control for CycD1 (1:1000; Sigma-Aldrich, Saint Quentin, France), Rb (1:1000; Cell Signaling Technology, Beverly, MA, USA) and RbS807/811ph (1:1000; Cell Signaling Technology, Beverly, MA, USA), and β-actin (1:1000; Cell Signaling Technology, Beverly, MA, USA) was used as an internal control for H4K8Ac and H3K18Ac (1:1000; Cell Signaling Technology, Beverly, MA, USA).

### 4.5. Tissue Printing

Apical root fragments (approx. 1.5 cm-long) were dissected longitudinally and blotted onto a nitrocellulose membrane in accordance with [8]. The procedure performed was similar to [4]. Anti-cyclin D1 (anti-CycD1;1:1000; Sigma-Aldrich, Saint Quentin, France) were used as primary antibodies together with secondary antibodies conjugated with alkaline phosphatase. The induction of color reaction was performed for 10 min with substrates for alkaline phosphatase—NBT and BCIP (nitro blue tetrazolium and 5-bromo-5-chloro-3-indolyl phosphate, respectively) in a buffer (100 mM Tris, pH 9.5; 100 mM NaCl; 5 mM/Mg Cl_2_). Prints were made using Stemi 2000C microscope (Zeiss, Jena, Germany), images were acquired with AxioCam ERc5s CCD camera (Zeiss, Jena, Germany).

### 4.6. Immunocytochemistry

Immunocytochemical procedures were performed similarly as described by [4,14,77]. 1.5 mm long apical parts of *V. faba* roots were fixed in PBS-buffered 3.7% paraformaldehyde (45 min, 18 °C), washed in PBS several times, and put into a digesting solution containing 2.5% pectinase (Fluka, Munich, Germany), 2.5% cellulose (Onozuka R-10; Serva, BIOKOM, Janki-Warsaw, Poland), and 2.5% pectolyase (Sigma, St. Louis, MO, United States), buffered with citric acid (pH 5.0; 45 min; 37 °C). After digestion, root tips were washed in PBS 3times, rinsed with distilled water, squash-flattened onto Super Frost Plus glass slides (Menzel-Gläser, Merck, Darmstadt, Germany), and air-dried. Slides prepared in that manner were next pretreated with 5% BSA containing 0.5% Triton X-100 (60 min, 20 °C) and incubated overnight (4 °C in a humidified environment) with a primary antibody (raised against Rb, RbSer807/811ph, H4K8Ac, and H3K18Ac, respectively) produced by rabbits (Cell Signaling Technology, Beverly, MA, USA, at dilution of 1:500). The slides were again washed three times after overnight incubation with PBT and next incubated with the secondary anti-rabbit IgG AlexaFluor 488 (Cell Signaling Technology, Beverly, MA, USA, at dilution of 1:1000) in PBT (2h, room temperature in the dark). Lastly, samples were washed with PBT and PBS (2 times, 5 min each). Additional DAPI staining of nuclear DNA (0.4 µg/mL) was also performed. After washing with PBS, the slides were air-dried and seated in mounting media (Vectashield, Vector Laboratories, Burlingame, CA, United States). Observations were made with AxioImager.A1 fluorescence microscope (Zeiss, Jena, Germany).

### 4.7. Image Analysis and 3D Modeling

Unless said otherwise, images were captured using AxioCam ERc5s CCD camera (Zeiss, Jena, Germany). Quantitative image analysis was performed on unedited images, converted to 8-bit grayscale in Fiji—an Open Source platform [78] based on *ImageJ* software (NIH and LOCI, University of Wisconsin, Madison, WI, United States). Post-analytic image processing was performed in Affinity Photo 18.5 and Affinity Designer 18.5 (Serif Europe Ltd., Nottingham, England). 3D models of nuclei and protein activity were prepared in Blender 2.9.1 (Blender Foundation, Amsterdam, The Netherlands), based on the microscope images of real samples.

### 4.8. Statistical Analysis

All statistical analyses were performed using Statistica 13.3 PL (Statsoft INC, Tulsa, OK, USA). The measured data were presented as mean ± SD (bar and whiskers plots) or raw data (scatterplots). Group differences were assessed by a one-way ANOVA and post-hoc analysis by Tukey’s test. Correlation coefficients r and r^2^ were calculated for the 2D scatterplots. Statistical significances were analyzed at *p* > 0.05.

## 5. Conclusions

Replication stress possesses a threat not only to the integrity of the genome itself, but also to the epigenetic modifications, especially within the heterochromatin area, which is usually under-replicated after the induction of replication arrest. Prolonged inhibition of fork progression interferes with the recycling of histones as well as the epigenetic marking of new ones and may lead to a loss of identity in new cells. Our findings suggest that mechanisms that respond to replication stress in root meristem cells of *V. faba* act in an orderly and precise manner. Even though the overall pRb activity is diminished, the activity profiles characteristic to the subsequent stages of replication are still clearly visible. One can only observe local, specific alterations, most probably related to DNA-damage cell response pathways or replication perturbations that need to be addressed. Facultative heterochromatin areas, especially in the perinucleolar region, seem to play a key role in this response as pRb activity is, at the very least, maintained within this area, and the number of nucleolus-active cells is increased after HU and PCC induction.

## Data Availability

Not applicable.

## Supplementary Materials

The following are available online at https://www.mdpi.com/article/10.3390/ijms22094572/s1.

## Abbreviations

ATR	ataxia telangiectasia and Rad3-related kinase
CF	caffeine
dNTPs	deoxyribonucleoside triphosphates
EC	euchromatin
HC	heterochromatin
HU	hydroxyurea
PCC	premature chromosome condensation
pRb	retinoblastoma protein
RNR	ribonucleotide reductase
