# Supplementary material for: Hydroxyurea and Caffeine Impact pRb-like Protein-Dependent Chromatin Architecture Profiles in Interphase Cells of Vicia faba
Source: Int J Mol Sci. 2021 Apr 27;22(9):4572. doi: 10.3390/ijms22094572 (PMC8123844; doi:10.3390/ijms22094572)
Supplement: Supplementary file 1 [file ijms-22-04572-s001.zip › ijms-1164861-supplementary.pdf]

Hydroxyurea and Caffeine Impact pRb-like Protein-Dependent Chromatin Architecture Profiles in Interphase Cells of *Vicia Faba*

Marcelina W. Musiałek [1], Joanna Deckert [2] and Dorota Rybaczek [1,*]

[1] Department of Cytophysiology, Institute of Experimental Biology, Faculty of Biology and Environmental Protection, University of Lodz, Pomorska 141/143, 90-236 Lodz, Poland; marcelina.musialek@edu.uni.lodz.pl (M.W.M.); dorota.rybaczek@biol.uni.lodz.pl (D.R.)

[2] Department of Plant Ecophysiology, Institute of Experimental Biology, Faculty of Biology, Adam Mickiewicz University Poznań, Uniwersytetu Poznańskiego 6, 61-614 Poznań, Poland; Joanna.Deckert@amu.edu.pl (J.D.)

* Correspondence: dorota.rybaczek@biol.uni.lodz.pl; Tel.: +48-42-635-47-35 (D.R.)

Supplementary results

Supplementary Figure S1. The general descriptors of the intensity of RbS807/811ph within nuclei. **A**. The significant differences betweenmean intensity (**a1**), standard deviation of intensity (**a2**), and maximum intensity (**a3**) between the control, HU, and PCC. Maximum intensity is a parameter describing the activity of singular pRb foci, while mean intensity describes the overall nucleus activity of the protein and thus should not be mistaken. Hydroxyurea (HU) and PCC induction result in the decrease of pRb activity (both general and foci-related) and in the decrease in the value of standard deviation, which implies that the activity of singular foci is similar to the general luminescence of the nucleus. **B**. The histograms show the mean activity of pRb in control (**b1**), HU (**b2**), and PCC (**b3**) series, shown as the lightness from 0 to 255, similarly as described in Figure 1. Under normal conditions, most cells show the general activity around 155. Hydroxyurea incubation shifts the majority of the cells to the lightness value of around 85, one 'step' lower than the control group (compare with the yellow dashed line). Induction of PCC shifts this value one another 'step' further to the left, to approximately 60 (as shown by the red dashed line). **C**. The histograms of max activity in the nuclei for control (**c1**), HU (**c2**), and PCC (**c3**) series. Maximum is usually two 'steps' higher than compared to the mean activity, but it also follows the same pattern in cells subjected to replication stress (yellow dashed line) and PCC induction (red dashed line). The differences were assessed by one-way ANOVA followed by post-hoc (Tukey's) test at the significance level of $p = 0.05$.

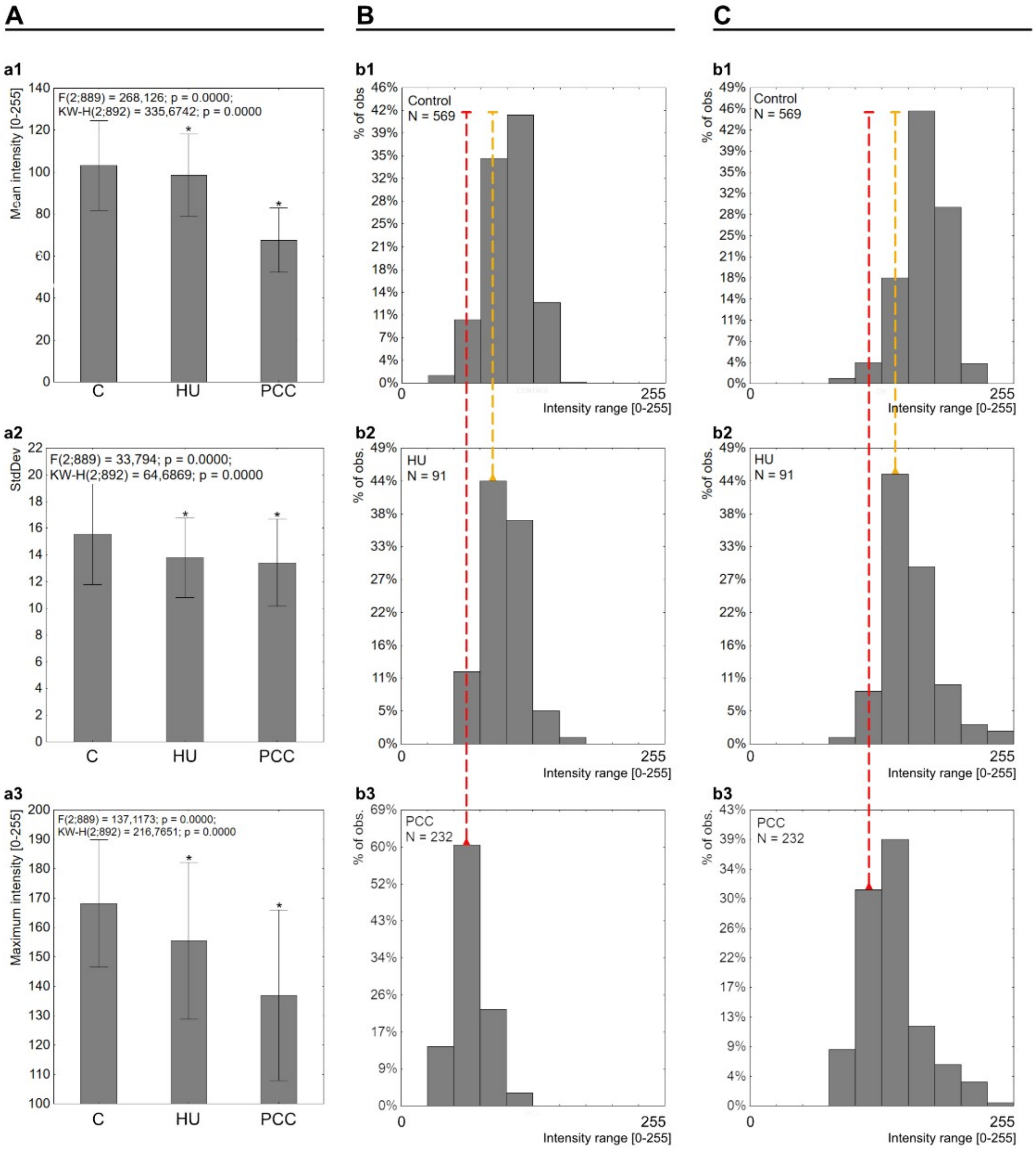

A
B
C
a1
F(2;889) = 268,126; p = 0.0000;
KW-H(2;892) = 335,6742; p = 0.0000
Mean intensity [0-255]
a2
F(2;889) = 33,794; p = 0.0000;
KW-H(2;892) = 64,6869; p = 0.0000
StdDev
a3
F(2;889) = 137,1173; p = 0.0000;
KW-H(2;892) = 216,7651; p = 0.0000
Maximum intensity [0-255]
C
HU
PCC
b1
Control
N = 569
b2
HU
N = 91
b3
PCC
N = 232
% of obs.
Intensity range [0-255]

Supplementary Figure S2. The main differences between the appearance of pRb foci as compared between the control, HU, and PCC. **A.** The bar charts displaying the mean differences in the number of foci (**a1**), their average size (**a2**), total area measured as the number of pixels (**a3**), and percent of nucleus area covered by active foci (**a4**). The stars mark the significant differences as measured against the control series. The whiskers show the mean value ± 0.5 standard deviation. **B, C, D,** and **E.** The scatterplots based on raw data, the correlations analyzed are as shown on the axes. In control cells (**b1–e1**), the total foci area correlates with the number of foci (**b1**), but the correlation between their average size and number is significantly lower (**c1**), which indicates that the total nucleus coverage is based primarily on the number of active spots of pRb rather than their size. Hydroxyurea-treated cells show, again, lower pRb activity, the correlation strengths, however, remain similar to the control. These data indicate that cells subjected to replication stress display a proper Rb-related stress response mechanism that prevents cell replication without inducing any aberrations. According to these data, PCC induction (**b3–e3**) alters the correlations between analyzed parameters suggesting that some areas may experience extremely high (or extremely low) instances of pRb activity. The red dashed rounded rectangles mark the visible differences between the characteristics of foci in PCC-induced cells.

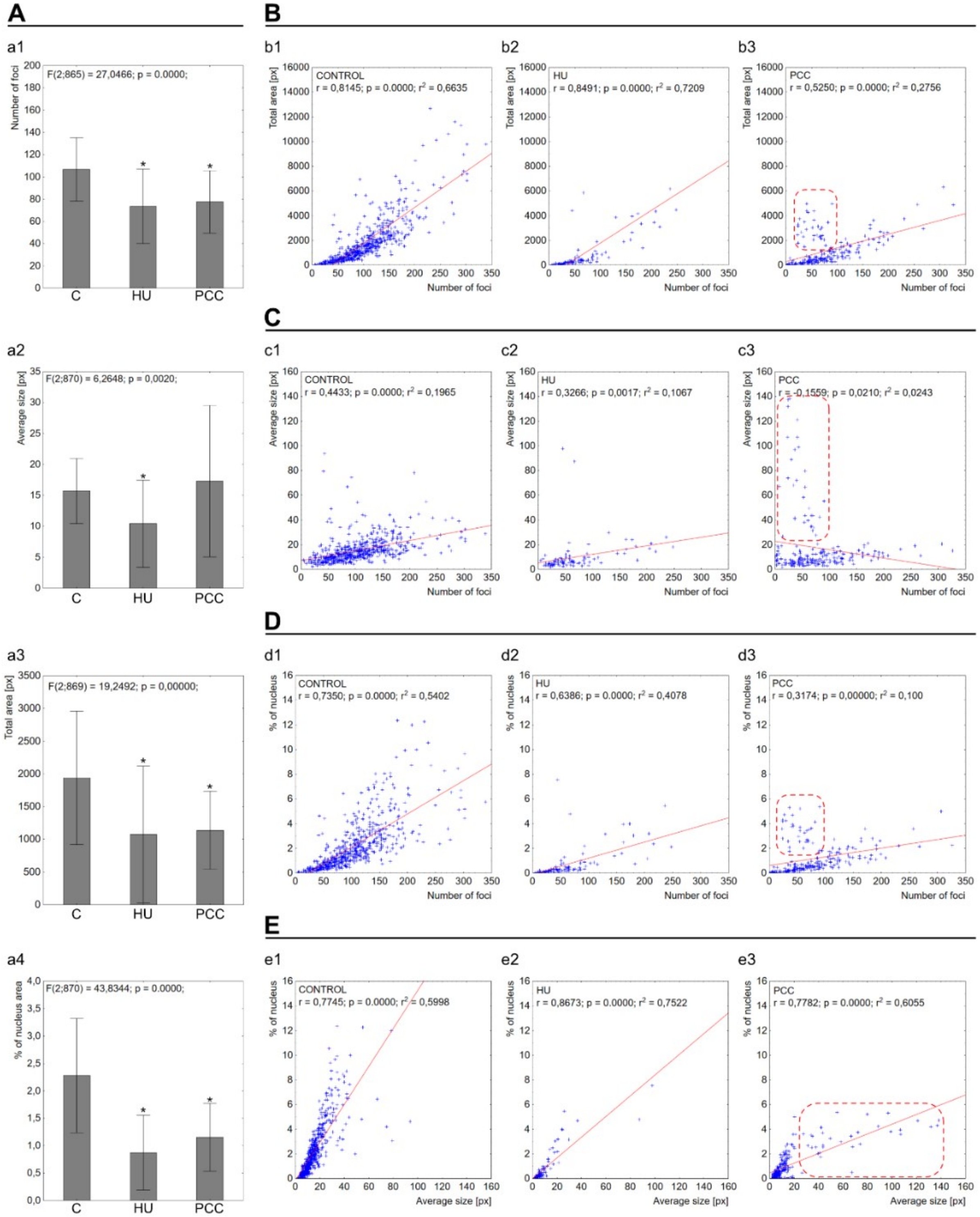
A
B
a1
F(2;865) = 27,0466; p = 0.0000;
Number of foci
C
HU
PCC
b1
CONTROL
r = 0,8145; p = 0.0000; r2 = 0,6635
Total area [px]
Number of foci
b2
HU
r = 0,8491; p = 0.0000; r2 = 0,7209
b3
PCC
r = 0,5250; p = 0.0000; r2 = 0,2756
C
a2
F(2;870) = 6,2648; p = 0,0020;
Average size [px]
c1
CONTROL
r = 0,4433; p = 0.0000; r2 = 0,1965
c2
HU
r = 0,3266; p = 0,0017; r2 = 0,1067
c3
PCC
r = -0,1559; p = 0,0210; r2 = 0,0243
D
a3
F(2;869) = 19,2492; p = 0,00000;
Total area [px]
d1
CONTROL
r = 0,7350; p = 0.0000; r2 = 0,5402
% of nucleus
d2
HU
r = 0,6386; p = 0.0000; r2 = 0,4078
d3
PCC
r = 0,3174; p = 0,00000; r2 = 0,100
E
a4
F(2;870) = 43,8344; p = 0.0000;
% of nucleus area
e1
CONTROL
r = 0,7745; p = 0.0000; r2 = 0,5998
Average size [px]
e2
HU
r = 0,8673; p = 0.0000; r2 = 0,7522
e3
PCC
r = 0,7782; p = 0.0000; r2 = 0,6055

Supplementary Figure S3. Statistical analysis of pRb activity profiles. **A.** The mean intensity of nucleus (**a1**, described as explained earlier in Figure 1), the number of foci (**a2**), their average size (**a3**, measured in pixels), total area (**a4**, also measured in pixels), and percent of nucleus area covered by pRb activity (**a5**) are five variables used to measure and describe pRb activity broken down into the particular activity types. The star (*) shows significant differences between Type 1 and other types of labeling. The black triangle (▲) shows significant differences between Type 2 and other types. The black circle (●) shows significant differences between Type 2a and other types. The white circle (○) shows significant differences between Type 2b and other types. The black rhombus (or diamond) shows significant differences between Type 3 and other types of labeling. **B.** The scatterplots of correlation between mean pRb activity in nucleus and the average size of pRb foci for each individual activity profile. **C.** The scatterplots of correlation between the percent of nucleus area covered by pRb activity and the number of foci in the nucleus. **D.** The scatterplots of correlation between the average size of pRb foci and their number. **E.** The in-depth analysis of mean intensity describing general pRb activity in the nucleus was measured separately for the individual activity type and divided into experimental series. **F.** The in-depth analysis of the percent of nucleus area covered by pRb activity measured separately for the individual type and divided into experimental series. The stars in E and F show significant differences between the control and given groups. The axis ranges for **B**, **C**, **D**, **E,** and **F**. were aligned thus they all show the same interval for corresponding variables. The significant differences for **A**, **E,** and **F** were assessed by one-way ANOVA followed by post-hoc (Tukey's) test at the significance level of p = 0.05. The r coefficient was calculated for each individual scatterplot and analyzed at the significance level of p = 0.05.

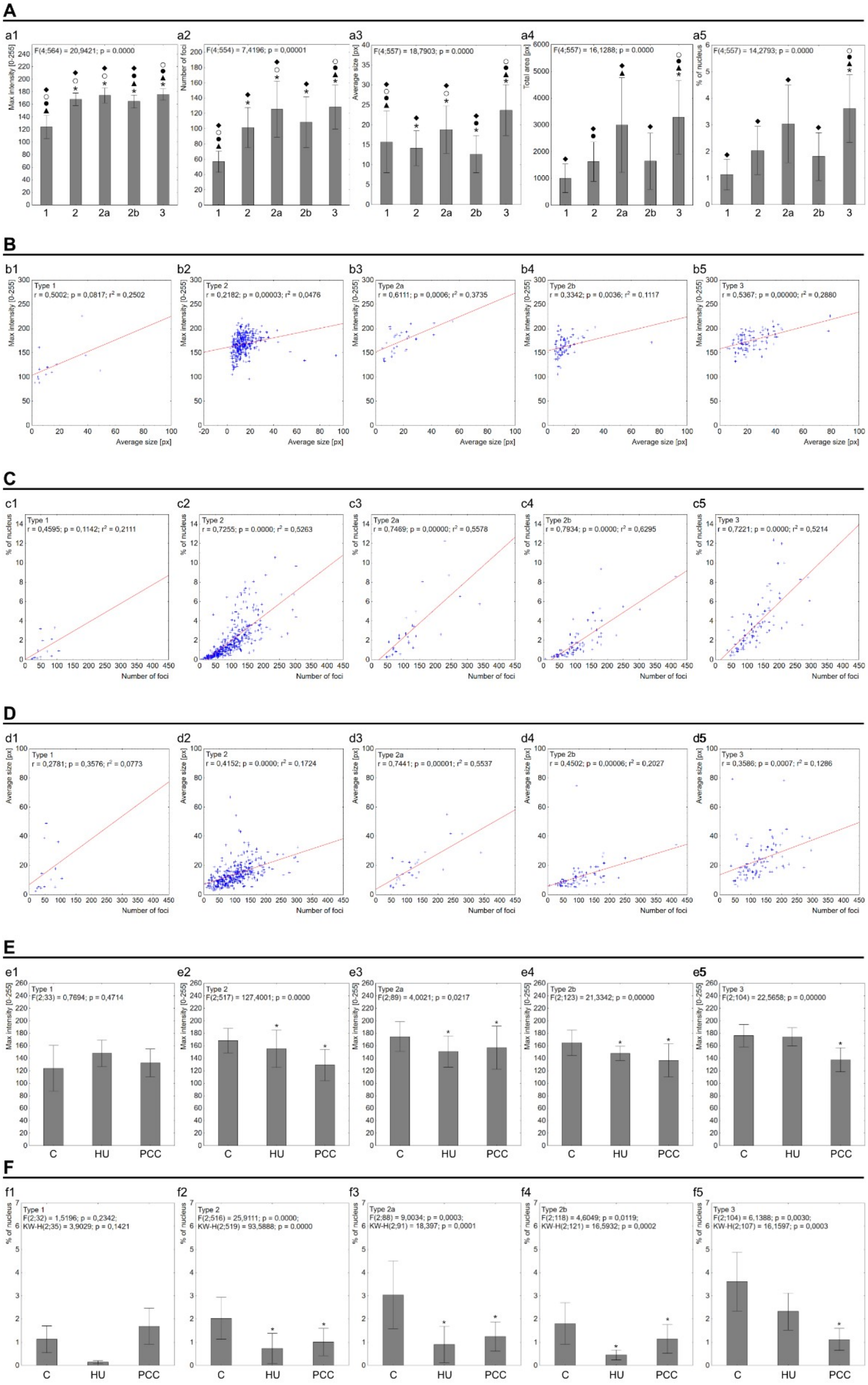
A
a1
F(4;564) = 20,9421; p = 0,0000
Max intensity [0-255]
a2
F(4;554) = 7,4196; p = 0,00001
Number of foci
a3
F(4;557) = 18,7903; p = 0,0000
Average size [px]
a4
F(4;557) = 16,1288; p = 0,0000
Total area [px]
a5
F(4;557) = 14,2793; p = 0,0000
% of nucleus
1
2
2a
2b
3
B
b1
Type 1
r = 0,5002; p = 0,0817; r² = 0,2502
b2
Type 2
r = 0,2182; p = 0,00003; r² = 0,0476
b3
Type 2a
r = 0,6111; p = 0,0006; r² = 0,3735
b4
Type 2b
r = 0,3342; p = 0,0036; r² = 0,1117
b5
Type 3
r = 0,5367; p = 0,00000; r² = 0,2880
Average size [px]
C
c1
Type 1
r = 0,4595; p = 0,1142; r² = 0,2111
c2
Type 2
r = 0,7255; p = 0,0000; r² = 0,5263
c3
Type 2a
r = 0,7469; p = 0,00000; r² = 0,5578
c4
Type 2b
r = 0,7934; p = 0,0000; r² = 0,6295
c5
Type 3
r = 0,7221; p = 0,0000; r² = 0,5214
Number of foci
D
d1
Type 1
r = 0,2781; p = 0,3576; r² = 0,0773
d2
Type 2
r = 0,4152; p = 0,0000; r² = 0,1724
d3
Type 2a
r = 0,7441; p = 0,00001; r² = 0,5537
d4
Type 2b
r = 0,4502; p = 0,00006; r² = 0,2027
d5
Type 3
r = 0,3586; p = 0,0007; r² = 0,1286
E
e1
Type 1
F(2;33) = 0,7694; p = 0,4714
e2
Type 2
F(2;517) = 127,4001; p = 0,0000
e3
Type 2a
F(2;89) = 4,0021; p = 0,0217
e4
Type 2b
F(2;123) = 21,3342; p = 0,00000
e5
Type 3
F(2;104) = 22,5658; p = 0,00000
C
HU
PCC
F
f1
Type 1
F(2;32) = 1,5196; p = 0,2342;
KW-H(2;35) = 3,9029; p = 0,1421
f2
Type 2
F(2;516) = 25,9111; p = 0,0000;
KW-H(2;519) = 93,5888; p = 0,0000
f3
Type 2a
F(2;88) = 9,0034; p = 0,0003;
KW-H(2;91) = 18,397; p = 0,0001
f4
Type 2b
F(2;118) = 4,6049; p = 0,0119;
KW-H(2;121) = 16,5932; p = 0,0002
f5
Type 3
F(2;104) = 6,1388; p = 0,0030;
KW-H(2;107) = 16,1597; p = 0,0003
